# A Method for Measurement of Nonlinearity of Laser Interferometer Based on Optical Frequency Tuning

**DOI:** 10.3390/s17122721

**Published:** 2017-11-24

**Authors:** Zhenyu Zhu, Xing Fu, Dongmei Ren, Yu Wan, Ji Wang

**Affiliations:** 1 College of Precision Instrument and Opto-Electronics Engineering, Tianjin University, Tianjin 300072, China; xingfu@tju.edu.cn (X.F.); wangji@cimm.com.cn (J.W.); 2 Changcheng Institute of Metrology & Measurement, Beijing, 100095, China; rendongmei@cimm.com.cn (D.R.); wanyu@cimm.com.cn (Y.W.)

**Keywords:** interferometry, frequency tuning, nonlinearity, measurement

## Abstract

A method for measuring the nonlinearity of laser interferometer using optical frequency tuning technique is presented in this paper. The basic principle of this method is to make the fractional part of an interference fringe change by tuning the laser frequency and determining the nonlinearity of interferometer by comparing the fractional fringe change measured by the interferometer to that calculated from the laser frequency change. An experimental interferometric system with a wavelength tunable laser source is set up and the nonlinearity of the interferometer is measured. Since it does not require the precise displacement mechanism to produce the optical path difference change, this method is more convenient to use and may achieve a higher accuracy than the conventional measurement methods. The nonlinearity of the arbitrary interferometric phase can be measured by changing the laser frequency with this method. Experiments results have shown that the repeatability of nonlinearity measurement is less than 0.2 nm. This method can be applied to interferometry-based high precision dimensional measurements, such as coordinate measurement and displacement sensor calibration.

## 1. Introduction

Laser interferometers have been extensively used in high precision displacement measurements [1,2,3,4,5,6]. With the development of science and technology, the requirements for measurement accuracy of laser interferometers have become higher and higher. For some small displacement measurement, the measurement accuracy has increased from tens of nanometers to several nanometers or even sub nanometer level [7,8,9]. However, the further improvement of measurement accuracy of interferometer may be limited by the nonlinearity of interferometer, which is caused by the interferometer structure and the nonlinearity of optical and electrical components [10,11,12,13,14]. For some precision displacement measurements, especially the measurement with an uncertainty of the same level as the nonlinearity, the nonlinear error must be corrected. Generally, the nonlinearity of laser interferometer is suppressed by hardware control and correction method to get more accurate measurement results.

Since the laser interferometric measurement has a characteristic that its results are accurate at integer interference periods, the nonlinearity of laser interferometer can be determined according to the measurement results at the points of integer periods by using a linear regression method. There are many methods for the correction of interferometer nonlinearity [5,12,15,16,17,18,19], such as the Heydemann correction method, the frequency domain analysis method and the linear fitting method. In these methods, the displacement produced by a high precision displacement stage is measured by the laser interferometer to obtain a group of redundant measurement data. Then, the data are fitted by least squares or other methods to find the optimal solution. The nonlinearity of laser interferometer can be determined by the difference between the measurement results and the fitting curve. The nonlinearity can be obtained from measurement results in a single or multiple interference periods according to its periodical characteristics. In practical measurements, the nonlinearity of interferometer can be corrected using the previous optimal estimation at different measurement points. Although these methods can reduce the nonlinear error in measurement results to some extent, they have a common problem that the measurement mirror of interferometers must move. Thus, the accuracy of nonlinearity correction with these methods are limited by the accuracy of displacement mechanism and sampling length.

A method for measurement of nonlinearity based on laser frequency tuning is presented in this paper. By this method, the nonlinearity is determined by comparing the actual measured phase of the interference signal to the ideal phase obtained by changing the laser frequency at every measurement point. The advantage of this new nonlinearity measurement method is that it eliminates the influence of displacement mechanism and thus may achieve higher measurement accuracy. This method can be used for compensation of interferometer nonlinearity in high precision measurements.

## 2. Laser Interferometer 

A laser interferometer can be constructed in many ways according to different measurement requirements, such as single frequency interferometer, dual frequency interferometer, differential interferometer, common optical path interferometer, equal optical path difference interferometer, and so on [1,2,20,21]. No matter in what form, a two-beam interferometer always measures the change of optical path length difference between its measurement arm and the reference arm to determine the displacement.

**Figure 1:**
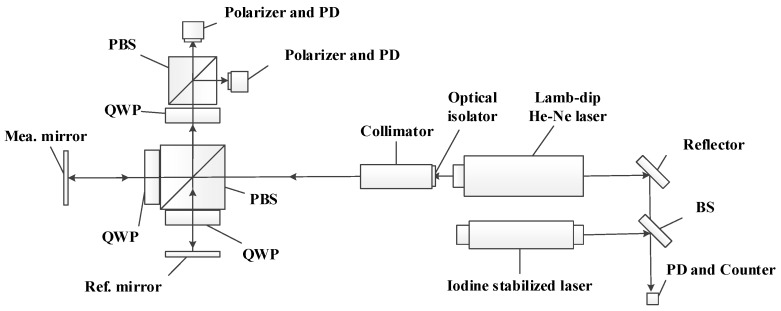
Diagram of interferometer for investigating the nonlinearity measurement method.

A laser interferometer as shown in Figure 1 is taken as an example to illustrate the nonlinearity measurement method based on laser frequency tuning. The light beam from a He-Ne laser enters the interference system and is divided into the reference beam and the measurement beam by a beam splitter. The two beams are reflected by the reference mirror and measurement mirror, respectively, and meet at the beam splitter to form an interference signal. The displacement of the measurement mirror can be determined according to the interference signal. In order to determine the change of displacement direction, the signal is separated into two parts with a phase difference of 90° and detected by two photoelectric detectors, respectively [20,22,23,24].

**Figure 2:**
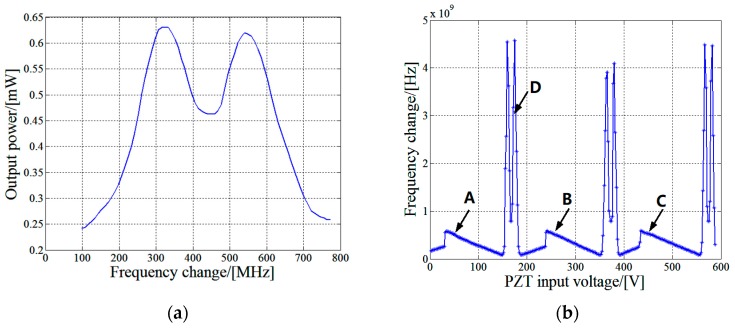
(**a**) Variation of Lamb dip laser power with frequency; (**b**) changes of laser frequency with input voltage of piezoelectric ceramic.

In this interferometer, the laser frequency tuning is realized by changing the cavity length of He-Ne laser using the piezoelectric ceramic that is designed for the control of Lamb dip laser frequency stabilization. A continuous frequency change of about 700 MHz can be obtained by this method. As shown in Figure 2a, the output power of the laser varies with frequency in the shape of “M”, and the frequency of the laser is usually stabilized at the central Lamb point. Figure 2b shows the frequency changes with the input voltage of the piezoelectric ceramic. The areas where the frequency changes continuously, such as A, B and C, are the effective working areas of the He-Ne laser. They can be used in the experiment of nonlinearity measurement. The area of laser mode change, such as D, should be avoided in the measurement. The frequency change of the He-Ne laser is determined by measuring the frequency difference between this laser and an iodine saturated absorption frequency-stabilized laser using a frequency counter.

**Figure 3:**
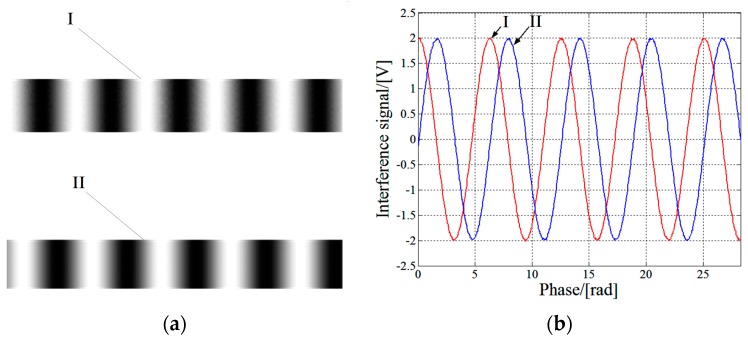
(**a**) Interference fringes; (**b**) interference signals.

The interference fringes and signals are shown in Figure 3. The two sets of interference fringes marked by I and II in Figure 3a correspond to the two signals in Figure 3b, respectively. The interference signals shown in Figure 3b are simulated ones used for illustration of the measurement principle. Due to the existence of nonlinearity, the orthogonal interference signals detected in the experiment may deviate from the standard sinusoidal wave form.

## 3. Principle of Nonlinearity Measurement

The basic principle of the nonlinearity measurement method based on laser frequency tuning is to make the fractional part of interference fringe change by tuning the laser frequency and determine the nonlinearity of interferometer by comparing the fractional fringe change measured by the interferometer to that calculated from laser frequency.

When the interferometer shown in Figure 1 is used for displacement measurement, the optical path difference between the measurement beam and the reference beam can be expressed as

(1) $$L=\frac{\lambda }{2}\left(N+\varepsilon \right),$$

where *L* is the optical path difference, λ is the laser wavelength, *N* is the integer part of interference fringe and ε is the fractional part of interference fringe. For convenience of description, the laser wavelengths mentioned below are wavelengths corrected for air refractive index.

When the nonlinearity of interferometer is measured using the laser frequency tuning method, the initial optical path difference is

(2) $$L=\frac{\lambda_{0}}{2}\left(N_{0}+\varepsilon_{0}\right),$$

where λ_0_ is the laser wavelength, *N*_0_ is the integer part and ε_0_ is the fractional part of interference fringe being measured at the initial state. Considering the relationship between the laser wavelength and frequency, Equation (2) can be expressed as

(3) $$L=\frac{c}{2n\nu_{0}}(N_{0}+\varepsilon_{0}),$$

where *c* is the light speed in vacuum, *n* is the refractive index of the media and *ν*_0_ is the laser frequency at initial state. When the laser frequency is tuned, the optical path difference becomes

(4) $$L=\frac{c}{2n\left(\nu_{0}+\Delta \nu_{i}\right)}\left(N_{i}+\varepsilon_{i}\right),$$

where Δ*ν_i_* is the frequency change during the tuning, *N_i_* is the integer part and ε*_i_* is the fractional part of interference fringe being measured at current state. By comparing Equations (3) and (4), the following equation is obtained

(5) $$N_{i}+\varepsilon_{i}=\left(N_{0}+\varepsilon_{0}\right)\left(1+\frac{\Delta \nu_{i}}{\nu_{0}}\right).$$

It is shown that all items on the right side of Equation (5) are known except Δ*ν_i_*. Thus, different *N_i_* and ε*_i_* can be obtained by adjusting Δ*ν_i_*.

Considering the fact that the integer part of the interference fringe can be measured accurately, the nonlinearity can be measured by the following procedures. First, the laser frequency is tuned to make the fractional part of an interference fringe be 0, namely, $\varepsilon_{0}=0$. Then, the laser frequency is continually changed to make the integer part of the interference fringe increase by 1 and the fractional part be 0 again, namely, $N_{i}=N_{0}+1$ and $\varepsilon_{i}=0$. Assuming that the frequency change that makes the interference fringe change by one period is Δ*ν*, the initial value of the integer part of the interference fringe can be determined by

(6) $$N_{0}=\frac{\nu_{0}}{\Delta \nu }.$$

Substituting Equation (6) and $\varepsilon_{0}=0$ into Equation (2), the actual optical path difference can be obtained. Then, the laser frequency is tuned. From Equation (5), the fractional part of interference fringe can be determined by

(7) $$\varepsilon_{i}=\left(1+\frac{\Delta \nu_{i}}{\nu_{0}}\right)N_{0}-N_{i}.$$

Assuming that the measurement range is smaller than one interference period, the integer part of interference fringe does not change, namely, $N_{i}=N_{0}=\frac{\nu_{0}}{\Delta \nu }$, and the actual value of fractional part of interference fringe at any measurement point in the single interference period can be determined by

(8) $$\varepsilon_{i}=\frac{\Delta \nu_{i}}{\Delta \nu }$$

It is shown in Equation (8) that the fractional part of interference fringe *ε_i_* is only related to the frequency change of an interference period Δ*ν* and the current frequency change Δ*ν_i_*.

The nonlinearity in an inference period can be obtained by comparing the fractional part of the interference fringe measured using the interferometer to the theoretical one calculated from Equation (8).

According to the measurement result of the interference phase and the phase calculated from the tuned frequency, the deviation of the measurement result from its exact value in an interference period can be determined, which shows the nonlinearity of the interferometer. The data can be recorded and used for automatic compensation of nonlinear error in the subsequent measurements. The benefits of the methods include (1) no mechanical movement of the mirror is necessary so that the error in movement control can be avoided; and (2) the measurement result of nonlinearity is traceable to the laser wavelength.

## 4. Experiments and Results

**Figure 4:**
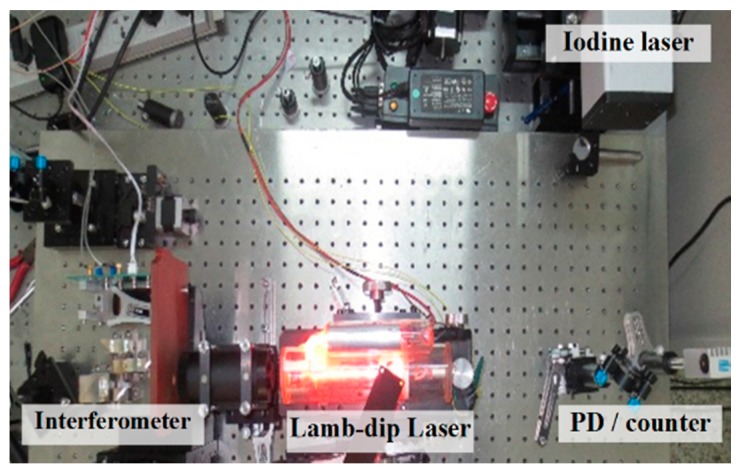
Experimental setup of nonlinearity measurement.

In order to test the performance of the nonlinearity measurement method based on laser frequency tuning, a laser interferometric system was setup, as shown in Figure 4. In the experiment, the measurement result of nonlinearity obtained by the laser frequency tuning method described above was compared to that obtained by the conventional linear fitting method. The experimental measurements were carried out in a laboratory, where the temperature is controlled to be (20 ± 0.2) °C.

Before measuring the nonlinearity of interferometer using optical frequency tuning method, the nonlinearity was measured using the linear fitting method, which is a commonly used method for nonlinearity measurement. In this experiment, the laser frequency was stabilized. The measurement mirror of interferometer was mounted on a P730 micro-displacement flexure stage produced by Physik Instrumente (PI) (Karlsruhe, Germany), which was driven by piezoelectric ceramic and equipped with a capacitive sensor. The stage had a motion range of 100 μm, a resolution of 0.2 nm, and an accuracy better than 1 nm.

**Figure 5:**
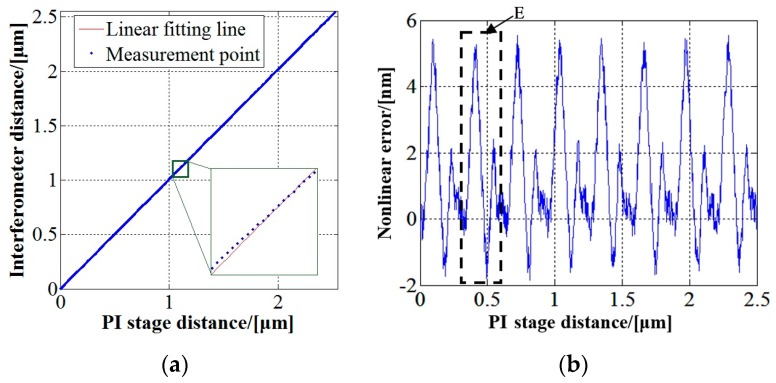
(**a**) Relationship between displacements measured by interferometer and micro-displacement stage; (**b**) measurement result of nonlinearity by linear fitting method.

When the measurement mirror moved with the stage, the measurement results of the interferometer and the displacement given by the micro-displacement stage were recorded synchronously. Figure 5a shows the relationship between the displacement measured by the interferometer and that given by the sensor on the stage over a range of (0–2.5) µm. A local zoom-in view is given to show the nonlinearity. In order to separate the nonlinear error from the measurement result, a linear fitting is made to the measurement points shown in Figure 5a. After removing the linear item, the deviation of the interferometer measurement result from the stage displacement is obtained, as shown in Figure 5b.

It can be seen obviously from Figure 5b that a periodic nonlinear error exists in the measurement result of the interferometer. Nonlinearity errors of different interferometers are different and random. For the specific system in Figure 4, the positive error is much greater than the negative one. The maximum value of the nonlinear error in this measurement is 5.6 nm. Shown in the dashed frame E is the nonlinear error in an interference period.

**Figure 6:**
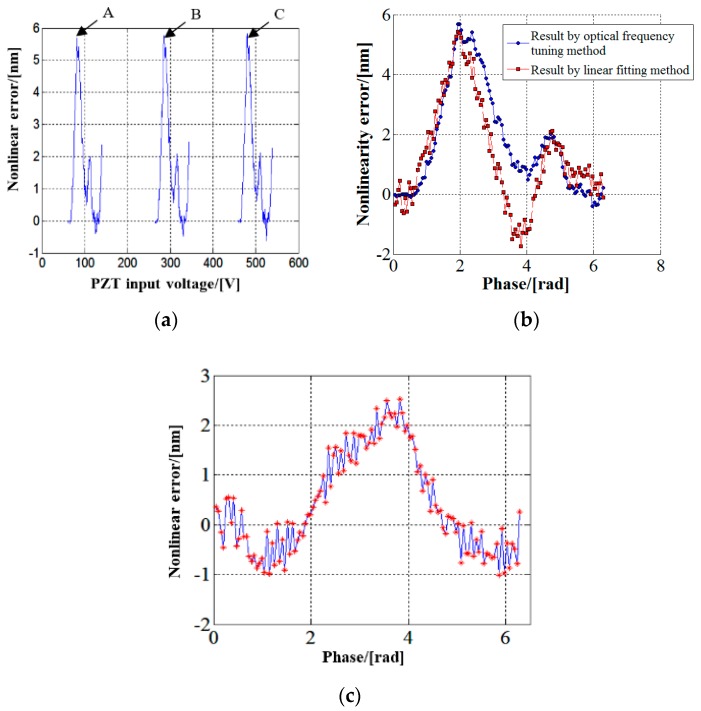
(**a**) Measurement result of nonlinearity by optical frequency tuning method; (**b**) comparison of the results of two methods; and (**c**) difference between measurement results of two methods.

When measuring the nonlinearity of interferometer using optical frequency tuning method, the laser frequency was tuned by adjusting the cavity length of the Lamb dip He-Ne laser. The laser cavity length was adjusted by controlling the voltage of the piezoelectric ceramic. In this experiment, the initial optical path difference between the measurement beam and reference beam of interferometer was about 300 mm. Considering that the initial laser frequency may locate outside the working area (A or B or C), the adjustment range of the piezoelectric ceramic input voltage should be no smaller than 200 V to cover a period between two mode change points, as shown in Figure 2b. The laser frequency was adjusted to the working area first and then the measurement process began. The laser frequency change was measured by comparison with the frequency of an iodine saturated absorption frequency-stabilized laser, which had a vacuum wavelength of 0.63299142704 μm and a relative wavelength uncertainty of 1 × 10^−10^ calibrated according to Reference [25]. The frequency difference between the He-Ne laser and the iodine frequency-stabilized laser as well as the temporal relation of the orthogonal interference signals were recorded synchronously. The zero point of orthogonal interference signals was calculated and taken as the starting point, and 120 measurement points were selected evenly in an interference period for nonlinearity measurement. The difference between the phase measured by the interferometer and that calculated from the laser frequency change at each measurement point was calculated and converted to displacement, which was taken as the measurement result of nonlinearity at this point. The measurement result of nonlinearity in the voltage scanning range is shown in Figure 6a. The three areas of A, B and C in this figure correspond to the areas of A, B and C in Figure 2b, respectively.

The measurement result of nonlinearity obtained by the optical frequency tuning method is compared to that obtained by the linear fitting method in a single period in Figure 6b. The difference between the measurement results of two methods is shown in Figure 6c. The maximum difference is 2.6 nm, which occurs at the position with a phase of 3.82 radians in the interference period.

**Figure 7:**
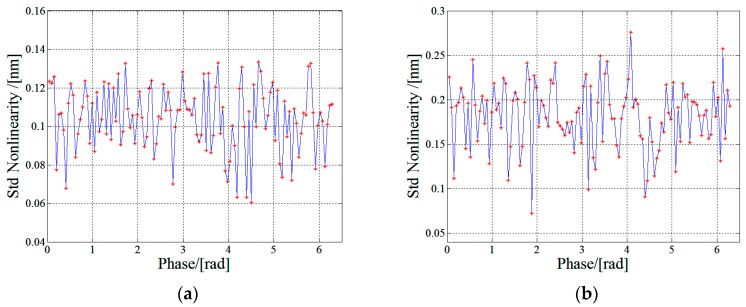
Standard deviation of nonlinearity measurement (**a**) with the proposed optical frequency tuning method and (**b**) with linear fitting method.

In order to characterize the repeatability of the new nonlinearity measurement method, the procedure of nonlinearity measurement by the optical frequency tuning method was repeated for 10 times. The experimental standard deviations of the 10 measurements on 120 points are calculated and the measurement result is shown in Figure 7a. It can be seen that the maximum value of the standard deviations on 120 points is less than 0.2 nm. As a comparison, the repeatability of the linear fitting method under the same condition is given in Figure 7b.

The experimental results have shown that the new nonlinearity measurement method discussed above agrees well with the conventional method and has a better repeatability. The reason for a higher repeatability may lie in the fact that the laser frequency tuning method does not need to mechanically move the target mirror to measure the nonlinearity of the system, so the errors in movement control can be avoided compared to the linear fitting method.

The key technique with this method is laser frequency tuning. With a frequency tunable laser and the laser frequency measurement system, this method can be applied to the nonlinearity measurement of various interferometers. Meanwhile, for the majority of laser interferometers with only a fixed frequency stabilized laser, an external system for wavelength tuning, such as an acousto-optic modulator, can be applied to realize the method. Other errors or limitations due to the introduction of external system may exist. Nevertheless, the proposed laser frequency tuning method has offered an accurate way of measuring the nonlinear error of the interferometer without the necessity of mechanical movement.

## 5. Conclusions

A novel method for the measurement of the nonlinearity of a homodyne interferometer based on optical frequency tuning is presented. Compared to the conventional methods of nonlinearity measurement, the novelty of this method lies in the fact that it can measure the nonlinearity of interferometer without moving the measurement mirror, and it can measure the nonlinearity of an arbitrary interferometric phase by changing the laser frequency. A laser interferometer is set up and the experiment of nonlinearity measurement is carried out. The experimental results show that the repeatability of nonlinearity measurement with this method is better than 0.2 nm. This method overcomes the limitation of conventional measurement methods, i.e. a precision displacement mechanism is required, so that it is more convenient to use and may further improve the measurement accuracy. Another advantage of this method is that the measurement results of nonlinearity are traceable to the laser wavelength. This method can be applied to interferometry-based high precision dimensional measurements, such as coordinate measurement and displacement sensor calibration. Limited by the laser frequency tuning range, the method currently requires that the measurement beam and reference beam have a certain initial optical length reference. With the advance in laser frequency tuning capability, the measurement accuracy and efficiency would be further improved with this method.
